# Mobile phone use, school electromagnetic field levels and related symptoms: a cross-sectional survey among 2150 high school students in Izmir

**DOI:** 10.1186/s12940-017-0257-x

**Published:** 2017-06-02

**Authors:** Raika Durusoy, Hür Hassoy, Ahmet Özkurt, Ali Osman Karababa

**Affiliations:** 1 Department of Public Health, Ege University Medical School, Ege Üniversitesi Tıp Fakültesi, Halk Sağlığı A.D., 35100 Bornova, Izmir, Turkey; 2 Department of Electrical and Electronics Engineering, Dokuz Eylül University Faculty of Engineering, Buca, Izmir, Turkey

**Keywords:** Adolescent, Mobile phone, Cell phone, Base stations, Electromagnetic field, Headache, Fatigue, Sleep disturbances, Depressive symptoms

## Abstract

**Background:**

Health outcomes of electromagnetic fields (EMF) from mobile phones and their base stations are of concern. Conducting multidisciplinary research, targeting children and exploring dose-response are recommended. Our objectives were to describe the mobile phone usage characteristics of high school students and to explore the association between mobile phone usage characteristics, high school EMF levels and self-reported symptoms.

**Methods:**

This cross-sectional study’s data were collected by a survey questionnaire and by measuring school EMF levels between November 2009 and April 2011. A sample size of 2530 was calculated from a total of 20,493 students in 26 high schools and 2150 (85.0%) were included in the analysis. The frequencies of 23 symptoms were questioned and analysed according to 16 different aspects of mobile phone use and school EMF levels, exploring also dose-response. School EMF levels were measured with Aaronia Spectran HF-4060 device. Chi square and trend tests were used for univariate and logistic regression was used for multivariate analyses.

**Results:**

Among participants, 2021 (94.0%) were using mobile phones and 129 (6.0%) were not. Among users, 49.4% were speaking <10 min and 52.2% were sending/receiving 75 or more messages per day. Headache, fatigue and sleep disturbances were observed respectively 1.90 (95% CI 1.30–2.77), 1.78 (1.21–2.63) and 1.53 (1.05–2.21) times more among mobile phone users. Dose-response relationships were observed especially for the number of calls per day, total duration of calls per day, total number of text messages per day, position and status of mobile phone at night and making calls while charging as exposures and headache, concentration difficulties, fatigue and sleep disturbances as general symptoms and warming of the ear and flushing as local symptoms.

**Conclusions:**

We found an association between mobile phone use and especially headache, concentration difficulties, fatigue, sleep disturbances and warming of the ear showing also dose-response. We have found limited associations between vicinity to base stations and some general symptoms; however, we did not find any association with school EMF levels. Decreasing the numbers of calls and messages, decreasing the duration of calls, using earphones, keeping the phone away from the head and body and similar precautions might decrease the frequencies or prevalence of the symptoms.

**Electronic supplementary material:**

The online version of this article (doi:10.1186/s12940-017-0257-x) contains supplementary material, which is available to authorized users.

## Background

The number of mobile-cellular telephone subscriptions was 7.216 billion worldwide, equivalent to 98.6 per 100 inhabitants in 2015 [1]. The same figure for Turkey was 73.7 million, equivalent to 93.5% of the total population or 106% of the population aged 10 years or older in 2016 [2]. With the widening use of mobile phones, health concerns have also increased. In PubMed, the yearly number of articles found with the search term “mobile phone” has steadily and logarithmically increased from <10 between 1992 and 1996 to 1675 in 2015, reaching a total of 9856 articles (PubMed search conducted on September 20, 2016).

The human body is lacking an organ or system to detect or sense radiofrequency electromagnetic fields (RF-EMF), in contrast to our eyes which detect visible light, another band of the electromagnetic spectrum, though cumulating literature about electromagnetic hypersensitivity (this term recently switching to idiopathic environmental intolerance to EMF) has opened a controversy on the presence of variations among human beings in a kind of ‘sense’ of electromagnetic fields, or a ‘sense’ of the changes occurring in the body under EMF, with many randomized provocation studies unable to prove a link between such sensitivity and RF-EMF [3–5]. However, our nervous system relies on electrical signals and physically it is known that electromagnetic fields have impacts on electrical conduction. Furthermore, studies have shown impacts of RF-EMF on many other mechanisms in biological tissues, classified under thermal and non-thermal effects [6–8].

There are studies finding a link between some symptoms and RF-EMF exposure. Chronic exposure to RF-EMF through the use of mobile phones or vicinity to their base stations has been linked to headache, fatigue, sleep disturbances, concentration difficulties, alterations in memory, warming of the ear and other symptoms [9–15]. The prevalence and frequency of some of these symptoms were increasing with increasing duration and number of calls per day or decreasing distance to base stations, which could be considered as signs of dose-response [12, 15–17]. These symptoms were termed as Non-Specific Health Symptoms in the literature [18]. Instead of these subjective complaints, other research teams have focused on objective assessments of the impacts of short-term exposure on the functioning of the nervous system and brain, with many different findings due to wide variations in outcomes and experimental designs [8, 19]. There are also studies showing an association with cognitive functions such as 7th grade students who speak more on their mobile phone responding more quickly but making more errors in cognitive tests [20].

Children and adolescents are considered more susceptible to the impacts of electromagnetic fields, due to continuing plasticity in their brain, thinner skull bones and their brains’ dielectric properties closer to soft tissues [21, 22]. Besides, as they start using the device earlier in life, in comparison with their parents who have met this technology in their adulthood, their cumulative exposure will be much higher to this agent classified as possibly carcinogenic to humans (Group 2B) by the IARC [23]. The 2006 WHO RF Research Agenda had put out a need for conducting research on all health outcomes among children and adolescents such as sleep, headaches and cognitive effects. The 2010 version of the WHO RF Research Agenda has expressed high-priority research needs on behavioural and neurological disorders among adolescents and recommends the investigation of dose-response relationships [24, 25]. The WHO had also recommended the design of studies characterizing the general population’s exposures from all RF sources by multidisciplinary research teams including epidemiologists, physicists and engineers [24]. The aims of our study were:

1. To describe the mobile phone usage characteristics of high school students in Bornova, Izmir
2. To investigate the frequencies of non-specific health symptoms that could be related with EMF among the students
3. To explore the association between mobile phone usage characteristics, high school EMF levels and these self-reported symptoms.

## Methods

The study includes a cross-sectional survey among high school students between 7 December 2009 and 15 April 2010 and electromagnetic field measurements at the same schools.

The target group of the study was students in all high schools in Bornova district of Izmir. Located on the Aegean coast of Turkey, Izmir is the third largest city of the country with 2.8 million metropolitan population in 2010. Among its metropolitan districts, Bornova had 419,070 inhabitants in 2010. In Turkey, there is a central high school entrance examination which enables students from all over the country to enter a high school located in any province according to the points they obtain. Among districts of Izmir, Bornova is unique with its high school education infrastructure, having two famous high schools favored from all around the country and also two high-capacity vocational high schools with more than 1700 students each.

There were totally 20,493 students attending 26 high schools in Bornova. We calculated the sample size as 2530 students using 50% prevalence, 3% error, a design effect of 2 and a non-response rate of 20%. We used a stratified clustered sampling scheme with stratification according to the size of the schools and classes forming the clusters. The average size of the classes was 29, thus we sampled 87 classes in a randomized systematic manner from a total of 704 classes to reach the sample size. Actually, there were 2466 students registered to these 87 classes, of whom 2240 (90.8% of actual number or 88.5% of the target sample size) were present in the classroom during data collection and all of them participated in the study.

We used a questionnaire comprising 84 questions: 11 on students’ socio-demographic characteristics, 24 on their mobile phone usage, one on the presence of a base station in their vicinity, 25 on their risk perception and 23 on symptom frequencies. Their risk perception was analyzed separately and presented elsewhere [26]. After the instructions of one of the researchers, the students filled out the questionnaires in about 20–25 min, with a researcher and a teacher assisting in the classroom. The questionnaire is presented as Additional file 1.

**Fig. 1:**
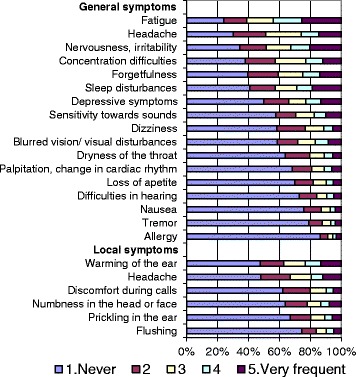
Frequencies of symptoms in the past one month (%)

The outcomes of this study were the presence and frequencies of 17 general and six local symptoms (Fig. 1) ever studied in the literature in relation to electromagnetic fields related to mobile phones or their base stations [14, 16, 17, 27, 28]. The general symptoms were questioned as “Have you ever had any of the following symptoms in the past one month?” and the local symptoms were questioned as “While you are having a call with mobile phone, do you experience any of the following symptoms on the side you use the mobile phone?”. For both groups of symptoms, the explanations “Please score each symptom between 1 and 5, ‘1’ meaning never and ‘5’ meaning very frequent” followed before the list of symptoms and their scores to mark. In the multivariate analyses, the general symptoms were dichotomized into the categories ever (responses 2–5) versus never (response 1) and the local symptoms among mobile phone users were dichotomized as frequent (responses 4 and 5) versus less (responses 1–3).

The exposures in this study were mobile phone usage characteristics, presence of a base station nearby and school EMF levels. Mobile phone usage characteristics were queried by 25 questions, ten as open-ended and 15 as closed-ended questions. As all calls and text messages sent and received require a first stronger connection to a base station, the total (not only outgoing but also incoming) numbers of these asked with open-ended questions and were later classified as in Tables 2, 3 and 4. Other variables increasing or decreasing exposure like earphones and mobile phones’ SAR values were also included in the study [29].

Mobile phone utilization and ownership; if owned, brand(s) and model(s) were questioned. Students who reported that they were using mobile phones were classified as current users, whether they owned one or not. The most important reason of their mobile phone ownership and their duration of mobile phone use in years were asked descriptively. The number of mobile calls per day, if not daily, per week was questioned and classified as <1 call, 1–4 calls, 5–9 calls and ≥10 calls per day for descriptive analysis and the last two categories were combined for multivariate analysis on general symptoms since there were only 95 students making ≥10 calls per day.

Whether they knew the SAR value of their mobile phone, if yes, the SAR value was questioned. However, only 12 among 31 students stating they knew the SAR could write down a value, and most were not consistent. We found the SAR values of the mobile phones of students from the producers’ web sites for students who reported their phone’s model. If not available there, we consulted web sites providing SAR lists. For students owning more than one mobile phone, the mean SAR value was used for analyses. With this method, SAR values could be determined for 1573 students (77.8% of mobile phone users).

The daily total duration of phone calls was asked as an open-ended question and classified as <5 min, 5–9 min., 10–30 min. and >30 min. Use of earphones was queried as the categories ‘yes, always’ , ‘yes, frequently’ , ‘yes, sometimes’ , ‘yes, rarely’ and ‘no, never’. These were regrouped into three categories for multivariate analyses: always, often/sometimes and rarely/never. Their total number of text messages per day was asked in number and then classified into the categories ‘no text message’ , <15, 15–74, 75–199, ≥200 text messages. Connection to the internet (3G) via mobile phone was queried and if they were connecting, students were expected to write down its duration in minutes per week. Few students were connecting to the internet as it was newly becoming available in Turkey during our data collection thus this exposure was categorized only as yes or no in the analyses.

We questioned the type of mobile service the students were using, whether prepaid or bill. We also asked their consumption as the monthly prepaid amount or monthly amount of the bill, their tariffs and use of promotions, if they used promotions, their type and content. Among these, we only used promotions as an exposure (yes/no), the others left descriptive.

The presence of a base station near their home and school was another self-reported exposure question, querying also its distance if present. The presence of a base station near home/school was classified as ‘no’ , ‘yes, near home’ , ‘yes, near school’ , ‘yes, near both home and school’ and ‘does not know’. Distance was categorized as ‘none or >300 m away’ and ‘≤300 m away’. The category of 1171 students not knowing about base stations nearby was excluded from the multivariate analyses about self-reported base station data.

Seven questions concerning the position and status of the mobile phone were added to the questionnaire after data collection had started and 667 students’ data could be completed by re-visits to schools, 187 could not be completed (among them, 178 were mobile phone users), thus in total 1963 students had responded to the full version of the questionnaire. We questioned whether they kept their mobile phone on their bedside at night, in which status (on/off) and at what distance. The responses to the last two questions formed our variable ‘status and position of mobile phone at night’ , categorized as off, on at ≥1 m, on at 25-99 cm, on at 0–24 cm and on, distance not specified. The 88 students in the last category with unspecified distance were excluded from multivariate analyses. We questioned where they carried their mobile phone during daytime and after categorizing the responses given to the free text other option, the categories were ‘on daily clothes or belt’ , ‘in bag’ , ‘in the pocket of overcoat or jacket’ , ‘combinations of the first three’ , ‘in the hand’ and ‘does not carry; leaves at home or in a furniture at school’ descriptively. As there were combinations of the first three, these were combined with ‘in the hand’ to form two categories of exposure as ‘does not carry; leaves at home or in a furniture at school’ and ‘on his clothes/in his pocket/bag’ for multivariate analyses. The status of their mobile phone while they are studying was questioned, with its distance as open-ended, and the responses were categorized into ‘off’, ‘on/ silent mode ≥1 m away’, ‘on/ silent mode <1 m away’ and ‘on/silent mode on himself’ with increasing exposure. The status (on/off) and use of mobile phone during charging (yes/no) were also asked.

As socio-demographic characteristics, the school’s name, type of school, program, grade and class were questioned. The students were classified into seven school types according to their school, and when in a multiple-program school, according to the program they were registered to. These types were normal high school with standard education, Anatolian high school with predominance in foreign language training, industrial vocational high school, trade vocational high school, girls’ technical high school, private high school and science high school with superior education in science and math.

The students’ birth date and gender were asked. Completed age was calculated using their birth date and the date when the questionnaire was applied. Income was questioned both subjectively (perceived income level as very good/good/medium/bad/very bad) and objectively. Subjective income was not used as 53.6% replied medium and 38.9% good. We divided the monthly total household income by the total number of people living in the house to calculate monthly income per capita. This variable was classified according to the starvation and hunger lines announced on 24 February 2010 (mid-data collection) for Turkey. These cut-offs were also converted to US dollars (USD) according to the Turkish Central Bank currency of the same day.

Father’s occupation, mother’s and father’s education were questioned descriptively. The six education categories were merged into three for presentation: Primary school or less (≤5 years), middle or high school (6–12 years) and university.

We revisited the participating schools between 8 February and 8 April 2011 and conducted electromagnetic field measurements using a Aaronia Spectran HF-4060 model portable high frequency spectrum analyzer device with frequency range 100 MHz - 6 GHz. We visited all indoor and outdoor environments of the schools to find the spots with highest total radiofrequency (RF) intensity indoors (school building) and outdoors (school garden). At these spots with highest RF, we measured the maximum value (peak hold) in dBm for total RF and separately for all of the frequency ranges available on the device: 0-1GHz (radio waves), 1-2GHz (3G), 2-3GHz, 3-4GHz, 4-5GHz, wireless (WLAN24), GSM900, GSM1800, UMTS and DECT, using the panning approach and recorded the values and their respective frequencies [30]. The measurements in dBm were converted into V/m using the device’s standard conversion table and students were classified according to the quartiles of the frequency ranges total RF power, 3G, GSM 900 and GSM 1800 separately for school building and school garden values. We also measured low frequency magnetic and electric fields at these spots and, if present around transformers, using Aaronia Spectran NF-3020 device with frequency range 10 Hz-400 kHz.

Among the 2240 respondents, 90 had not replied to any of the symptom frequency questions and were excluded from the study. Thus the analyses were conducted on 2150 students, equivalent to 85.0% of the target sample size or 102.0% of the minimum required sample size discarding non-response. We presented descriptive statistics as frequencies and percentages. We showed means with their standard deviations. Univariate analyses on factors associated with symptoms were conducted with the chi-square test for nominal and chi squared test for trend for ordinal exposure variables. The frequencies of 21 out of the 23 symptoms changed significantly according to gender (more among girls) and significant changes were also observed according to school types. Each general and local symptom was analyzed according to one mobile phone use predictor at a time, adjusted for gender and school type with multivariate logistic regression. In the multivariate analyses on general symptoms, the baseline category for all analyses were students not using mobile phones, with increasing exposure through the categories of the exposure variable. As the local symptom questions were on symptoms experienced during calls, they were compared among mobile phone users (*n* = 2021) and their reference categories were the first, least exposed situations of the exposure variable, represented with OR = 1 in the table.

As many symptoms’ ORs increased with especially increasing number and duration of calls, and as mobile phones are a nearer source of RF-EMF compared to base stations, the multivariate analyses on the presence of general symptoms associated to base stations and school EMF levels were adjusted for gender, school type and total duration of mobile calls per day classified into five categories as non-users, <5 min, 5–9 min., 10–30 min. and >30 min.

## Results

The mean age of the participating students was 15.6 ± 1.3. A description of the study population is presented in Table 1.

**Table 1:** A description of the study population (*n* = 2150)

Characteristic	Number	Percent
Gender		
Male	1027	47.8
Female	1121	52.2
Grade		
9th grade	738	34.3
10th grade	561	26.1
11th grade	459	21.3
12th grade	392	18.2
Type of high school		
Normal high school	558	26.0
Anatolian high school	494	23.0
Industrial vocational high school	489	22.7
Trade vocational high school	285	13.3
Girls’ technical high school	212	9.9
Private high school	76	3.5
Science high school	36	1.7
Monthly income per capita (*n* = 1539)		
Below the starvation line (<137 USD)	467	30.3
Between the starvation and poverty lines (137–347 USD)	742	48.2
Above the poverty line (>347 USD)	330	21.4
Mother’s education		
Primary school or less (≤5 years)	1025	47.9
Middle or high school (6–12 years)	788	36.8
University	328	15.3
Father’s education		
Primary school or less (≤5 years)	659	30.8
Middle or high school (6–12 years)	1015	47.4
University	466	21.8
Presence of a base station nearby		
No, none nearby	392	18.7
Yes, close to home	303	14.5
Yes, close to school	150	7.2
Yes, close to both home and school	79	3.8
Does not know	1171	55.9
Distance to base station (*n* = 924)		
None nearby or >300 m	496	53.7
≤ 300 m	428	46.3
School building RF quartiles		
1st quartile (≤0.602 V/m)	657	30.6
2nd quartile (0.603–0.850 V/m)	521	24.2
3rd quartile (0.851–1.51 V/m)	438	20.4
4th quartile (≥1.52 V/m)	534	24.8

Among the participant high school students, 94.0% (*n* = 2021) were using mobile phones and 91.4% (*n* = 1966) had his/her own mobile phone. The ratio of mobile phone users and owners were respectively 89.8% and 86.0% at 9th grade and 98.2% and 97.7% at 12th grade and both significantly increased with increasing school grade (Chi square for trend *p* < 0.001 for both). A higher proportion of girls were using mobile phones, as compared to boys (95.4% vs. 92.5, *p* = 0.005). Mobile phone use ratio was highest in science and private high schools (100.0%) and lowest in vocational high schools (88.8% in trade and 89.2% in industrial types, *p* < 0.001). Mobile phone use was lowest among students below the starvation line (86.9%), highest above the poverty line (99.1%) and 95.1% in-between (*p* < 0.001). Mobile phone use showed an increasing trend with increasing mother’s and father’s education (both *p* < 0.001). When questioned about the most important reason for mobile phone ownership, 80.7% (*n* = 1435) stated their family’s will to be in touch with them, 11.4% (*n* = 203) to communicate with friends more easily, 2.4% (*n* = 42) most of their friends having mobile phones, 2.3% (*n* = 41) their own will and 3.3% (*n* = 58) other reasons.

Students were using mobile phones since 4.1 ± 1.8 years. Only 31 students (1.4%) stated that they knew the SAR value of their mobile phone and among them, only nine could write down a SAR value. Participating students’ mobile phone usage characteristics are shown in Table 2. The promotions used by the students included mostly text message packages, the most frequent being a promotion of 5000 text messages per month stated by 539 students (26.7% of users).

**Table 2:** Mobile phone consumption characteristics of participating students (n, %)

Characteristic	n	% among users (*n* = 2021)	% among all (*n* = 2150)
Mobile phone use			
Does not use a mobile phone	129	-	6.0
Uses a mobile phone	2021	100.0	94.0
Number of calls per day (*n* = 1915)			
< 1 call	498	26.0	24.4
1–4 calls	1103	57.6	54.0
5–9 calls	219	11.4	10.7
≥ 10 calls	95	5.0	4.6
Total duration of calls per day			
< 5 min	583	31.1	29.1
5–9 min	344	18.3	17.2
10–30 min	715	38.1	35.6
> 30 min	234	12.5	11.7
Use of earphones during calls			
Yes, always	17	0.9	0.8
Yes, frequently/ sometimes	346	17.5	16.4
Yes, rarely	352	17.8	16.7
No, never	1265	63.9	60.0
Total number of text messages per day			
No text message	59	3.1	2.9
< 15	386	20.5	19.2
15–74	456	24.2	22.7
75–199	435	23.1	21.6
200+ text messages	548	29.1	27.2
Connecting to the internet via mobile phone			
No	1768	89.2	83.8
Yes	214	10.8	10.1
Use of promotions			
No	549	28.0	26.2
Yes	1414	72.0	67.6
Type of tariff plan			
Prepaid	1909	95.3	88.8
Bill	75	3.7	3.5
Both types	20	1.0	0.9
SAR value of mobile phone (*n* = 1573)			
< 0.50	165	10.5	9.7
0.50- < 0.75	697	44.3	41.0
0.75- < 1.00	473	30.1	27.8
≥ 1.00	238	15.1	14.0
Status and position of mobile phone at night			
Off	303	21.8	19.9
On, ≥1 m away	538	38.6	35.7
On, 25-99 cm away	261	18.8	17.2
On, 0–24 cm away	202	14.5	13.3
On, distance not specified	88	6.3	5.9
Position of phone during daytime			
On daily clothes or belt^a^	1114	61.6	51.8
In their bag	295	16.3	13.7
In the pocket of overcoat or jacket	223	12.3	10.4
Combinations of the three above	67	3.7	3.2
In the hand	24	1.3	1.1
Does not carry; leaves at home or in a furniture at school	86	4.8	4.4
Status of phone while studying			
Off	159	9.2	8.5
On/silent mode ≥ 1 m away	292	16.8	15.8
On/silent mode < 1 m away	111	6.4	6.0
On/silent mode on himself	1174	67.6	63.1
Charges phone in which mode			
Off	295	16.4	15.9
On	1503	83.6	78.6
Makes calls while charging			
No	864	47.5	45.2
Yes	954	52.5	49.3

^a^Only five students reported carrying on their belt

The frequencies of the symptoms reported for the past 1 month are shown in Fig. 1. Fatigue was the most commonly encountered general symptom, followed by headache and irritability. Allergy, tremor and nausea were the least frequent general symptoms. Among local symptoms during calls, warming of the ear and headache were most common and flushing the rarest. All of the general and local symptoms were significantly more prevalent among girls than boys, except hearing difficulties and allergy which had no significant difference according to gender.

The relation of the presence of general symptoms with mobile phone use characteristics are explored in Table 3 (three or more significant associations) and Additional file 2 (fewer associations or no association). Among general symptoms, headache, fatigue and sleep disturbances were the three symptoms significantly more reported by mobile phone users compared to non-users (ORs 1.90, 1.78 and 1.53, respectively, Table 3). Significant increases in the prevalence of headache, dizziness, concentration difficulties, fatigue, sleep disturbances, depressive symptoms and dryness of the throat were observed with increasing number of calls and increasing duration of calls per day (Table 3). The prevalence of visual disturbances and arrhythmia increased with increasing duration of calls per day (Additional file 2). Students who were always using earphones during calls did not have significantly elevated prevalence of symptoms, while students using earphones rarely or never significantly had higher prevalence of headache, concentration difficulties, fatigue and sleep disturbances compared to non-users and significant trends were observed according to their frequencies of earphone use. As for the students’ total number of text messages per day; headache, dizziness, concentration difficulties, fatigue, sleep disturbances, visual disturbances and depressive symptoms were more prevalent among students sending and receiving ≥200 text messages per day, compared to non-users, and increasing trends with increasing number of text messages per day were observed for these symptoms and arrhythmia (Table 3 and Additional file 2). More of the students using promotions had headache, concentration difficulties, fatigue and sleep disturbances compared to non-users and students not using promotions had in-between symptom prevalence, contributing to the trends observed in the first seven symptoms in Table 3. The odds ratios were increasing with increasing SAR values. Significant associations were found between the SAR value of mobile phone and headache, concentration difficulties, fatigue and sleep disturbances (Table 3).

**Table 3:**
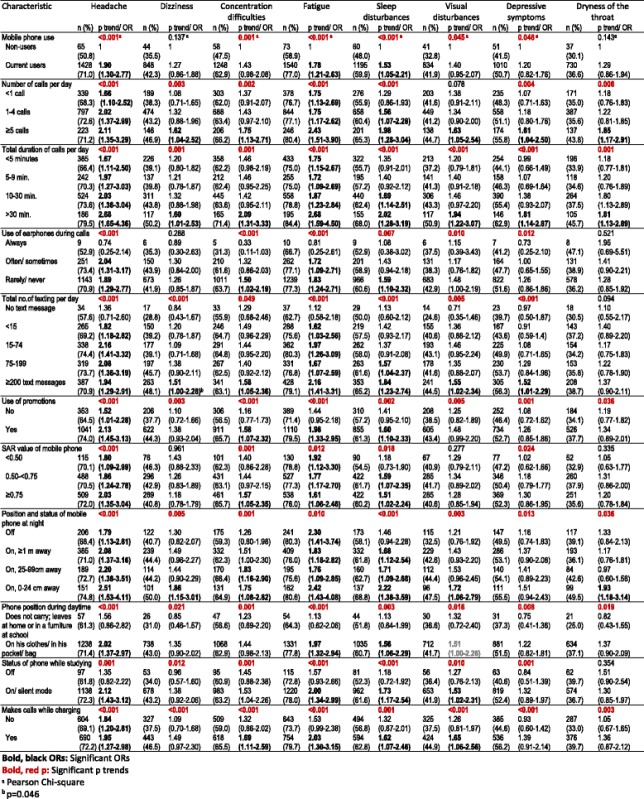
Presence of general symptoms with respect to mobile phone usage parameters; p trend, OR (adjusted for gender and school type) and 95% confidence intervals; non-users as the reference category of each analysis

Most of the general symptoms in Table 3 were associated with the position and status of mobile phone at night. ORs increased for all symptoms for students who kept their mobile phone switched on at 0–24 cm distance. Significant ORs were between 1.72 and 2.51 and only depressive symptoms’ ORs were insignificant. Students who carried the mobile phones on themselves and who kept their mobile phones switched on or in silent mode while studying had increased risk of headache, fatigue, sleep disturbances. Making calls while charging was significantly associated with headache, concentration difficulties, fatigue, sleep disturbances and visual disturbances. The ORs for the increased risk varied between 1.62 and 2.03 (Table 3).

Among the eight general symptoms not shown in Table 3, forgetfulness was observed 1.76 times more among students keeping their mobile phones on and at 25–99 cm at night, compared to non-users. Irritability was observed 1.58 times more among students making five or more calls per day and 1.78 times more among students speaking >30 min per day. Hearing difficulties were observed 0.64 times less among students using mobile phones with SAR ≥ 0.75 and 0.57 times less among students who do not make calls while charging the phone, both compared to non-users (Additional file 2). Among the general symptoms, five had no significant relationship with any of the independent variables: Tremor, nausea, loss of appetite, sensitivity towards sounds and allergy (Additional file 2).

Compared to non-users, charging the mobile phone in mode “off” and “on” were associated with 1.73 (1.08–2.76) and 1.89 (1.25–2.86) times increase in headache. Compared to non-users, charging the mobile phone in mode “on” was associated with 1.55 (1.02–2.35) times increase in concentration difficulties and 1.52 (1.01–2.30) times increase in sleep disturbances, while students charging their phone in mode “off” (*n* = 313) showed no increased risk for these two. Among exposure variables, there was no significant association between connection to the internet and general symptoms (data not presented in tables).

As for local symptoms, all were observed with significantly higher frequency with increasing number of calls and total duration of calls per day, compared to students making <1 call or speaking <5 min per day. Connection to the internet was associated with a 1.54 times increase in frequent headache as compared to mobile phone user students not connecting to the internet. Use of promotions was associated with a 1.29 times increased risk of frequent warming of the ear. Compared to students keeping their mobile phone off at night, the position of mobile phone kept on at night did not show a consistent significant pattern except flushing. Flushing risk was increasing with shortening distance. Making calls while charging was significantly associated with all six symptoms. The ORs for the increased risk varied between 1.33 and 1.95 (Table 4). No other significant association was found between the rest of the other exposure variables and local symptoms (data not shown).

**Table 4:**
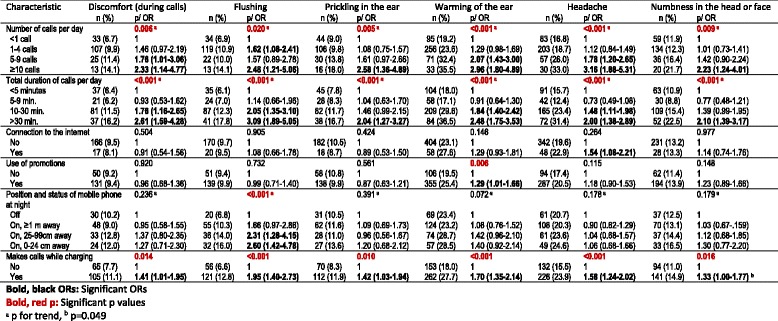
Local symptoms experienced on the ipsilateral side during mobile phone calls (frequencies 4 or 5 versus less or no) compared among users (*n* = 2021); ORs adjusted for gender and school type, reference categories are the first, represented with OR = 1

Compared to students reporting the absence of base stations near home or school, students reporting the presence of a base station near home experienced significantly more concentration difficulties, forgetfulness, visual disturbances, arrhythmia, sensitivity towards sounds, difficulties in hearing, with ORs varying between 1.44 and 1.58. For students reporting base stations near both home and school, the ORs were moderately increasing to 1.76 for sensitivity towards sounds and 2.03 for difficulties in hearing. The students who reported a base station less than 300 m from home or school had 1.56 times increased risk of visual disturbances, 1.37 times increased risk of arrhythmia, 1.43 times increased risk of sensitivity towards sounds, 1.76 times increased risk of difficulties in hearing. As for the electromagnetic field levels of the school buildings measured at RF, 3G, 900 MHz and 1800 MHz frequency bands, we found associations in the 3rd quartile of school building RF values and some of the symptoms such as nausea, dryness of the throat, sensitivity towards sounds, difficulties in hearing, allergy, as compared to the first quartile. There were also some associations between the school building 3G values and dryness of the throat, sensitivity towards sounds, difficulties in hearing, allergy symptoms. Additionally we found that school building 900 MHz values were in significant relation with tremor, dryness of the throat, sensitivity towards sounds symptoms. These associations did not show a consistent pattern. Some associations showed discrepancies. We found no significant relation between the electromagnetic values of the schools’ gardens and the health symptoms surveyed (Additional file 3).

## Discussion

The mobile phone ownership and utilization ratios were high among adolescents, who are considered as a risk group for electromagnetic field exposure. The students’ mobile phone ownership and utilization ratios were increasing with increasing grade, while even the ratios 89.8% and 86.0% of 9th grade students could be considered as high. The 91.5% mobile phone ownership ratio in our study was similar to the 91.4% found in a population-based survey conducted in 2006–2007 in Germany among 1508 adolescents aged 14–17 years but lower than 95.0% of the 14-year old group in a population-based survey conducted in 2005–2006 in Sweden [31, 32]. Our participants’ four-years mean duration of mobile phone use showed how early they had started using them and might be of concern for potential health risks in the future. Most of the students stated their family’s need to get in touch with them as the predominant reason for acquiring a mobile phone. As it was a self-reporting questionnaire, students might have marked this response even if their own wish to have a mobile phone were more important. In any case, so many parents’ will or final decision to buy them might be questionable and might show a need to inform parents on potential health risks to their children.

The students mostly used mobile phones for text messaging rather than voice calls. This pattern was also reflected in their preference to use prepaid tariffs and text message promotions. As the phone is at a greater distance from the body during texting, it might be considered better for health in terms of electromagnetic field exposure. However, their daily number of text messages sent and received are very high and might be linked to some orthopedic problems not questioned in this survey, since touch screens were not yet available and they had to push buttons multiple times to write letters. The lack of orthopaedic questions might be considered a limitation in detecting health impacts, though our scope was limited to EMF-related symptoms. With the advent of smart phones and Wi-Fi technology, this text messaging behaviour might have shifted to on-line texting, thus they could be more exposed to 2100 and 2400 MHz of 3G and Wi-Fi instead of 900 or 1800 MHz of 2G.

The students were unaware of their mobile phones’ SAR values, although sometimes youth might be considered better in following technology. We observed that the teachers in the same schools were also lacking knowledge about SAR. Probably there is a general lack of information in the public about SAR. The students were generally using mobile phones with high SAR values. A reason might be this lack of information while other reasons could be the market share of phones with high SAR values, the mobile phone producers’ disregard of the topic and the lack of information on SAR values in mobile phone advertisements.

Although six in 10 students never used earphones during calls, earphone use was higher than the ratio of 17.4% among Swedish adolescents having mobile phone access [33]. The use of this protective equipment was encouraged during training sessions held in six of the participating schools but wider dissemination of this information could be useful. Only 11% of the students were connecting to the internet via their mobile phone, as 3G communication started in 2009 in Turkey [34] and smart phones were also not available during the data collection period. Most of the students kept their mobile phones turned on, at their bedside during the night (some even under their pillows), a behavior increasing both the duration and intensity of exposure. The ratio of students keeping their mobile phones on themselves during daytime was also high.

Many of the symptoms previously found to have a link with mobile phones were also found to be associated with mobile phone use in this study. Compared to non-users, headache, fatigue and sleep disturbances were the three symptoms observed significantly more among mobile phone users.

In our study, dose-response relationships were more conspicuously observed for the number and total duration of calls per day in nine and 10 of the general symptoms, respectively, and for all of the six local symptoms. A survey of Swedish adolescents aged 15–19 years has found increasing and significant ORs with increasing duration of calls per day, categorized as 2–15 min and >15 min per day, for the eight symptoms allergy, asthma, hay fever, dizziness, headache, concentration difficulties, stress and tiredness among the 23 symptoms surveyed [33]. A study on medical students had found increasing prevalence of eight symptoms with increasing duration of calls per day, among the categories of <30, 30–60, 60–90 and >90 min per day, much higher durations than our group of adolescents [15]. In a population survey using personal dosimeters, headache was the only symptom observed 1.5 times more among adolescents in the 4th quartile of exposure, irritation among children and no association with nervousness, dizziness, concentration problems and fatigue. The position of the dosimeter on the contralateral upper arm and lack of night time or daytime sitting hours could be considered limitations of their evaluation [35]. Another provocation study has demonstrated a significant increase in headache after approximately 3 h of exposure, among 15 symptoms questioned [36]. In a one-year cohort study among college students, high text messaging was associated with prolonged stress among women and symptoms of depression among men [37]. Previously published reviews include several studies indicating possible causal relationship between exposure to EMF and the occurrence of these symptoms [38, 39].

The study among medical students had found a decrease in the prevalence of three symptoms in the last category of students using >90 min per day, although there is an increasing trend until the previous category [15]. We had observed a similar situation in some of our dose-response explorations, especially on general symptoms and the last category of students having 10 or more calls per day. This might either be linked to the fewer number of students in that category or the possibility of an adaptation or desensitization of the body under more intensive EMF signals, which could be explored in future studies. However, some associations were lost when the >10 calls per day category was merged with 5–10 calls, like tremor seen 2.39 times more among students talking 10 times or more per day.

A recent cohort study from Switzerland has found no association between far-field RF-EMF levels modelled or self-reported mobile phone use and symptoms scores. This study had explored a change in EMF exposure in 1 year and associated it with change in the total score of symptoms obtained from a scale and far-field EMF levels rely on a model [40]. Some of the symptoms in the scale might not be linked to EMF while others might be linked, as in our study, and a total score might mask these associations. Santini had showed that people living in the zone of 300 m from a base station complained significantly more often of some symptoms (till 300 m for tiredness, 200 m for headache, sleep disturbance, discomfort and 100 m for irritability, depression, loss of memory, dizziness, libido decrease). Another study on adults suggests a higher prevalence of insomnia among persons living in areas with higher exposure to electromagnetic fields where the number of radio antennas and cell towers was higher [41]. In the present study we found increased risks of visual disturbances, arrhythmia, sensitivity towards sounds, hearing difficulties in significant relation with proximity to base stations [17]. A reason for this difference may be the adolescents’ lack of precision in estimating distance to base stations. Another reason for being associated with fewer and different symptoms might be due to the radiation angle of the base stations (which is 120 degree like a flashlight). Even if the antennas are too close, the direction of radiation may be different due to the angle. The lack of a significant correlation between EMF measurements and distance to base stations from a study in Poland supports this argument. The same research had found no association between measurements at home and symptom frequency, while they had found associations between distance to base stations and the frequencies of headache and impaired memory [42]. In such evaluations, the participants’ own mobile phone usage might be a confounding factor especially for intensive users, since it is a much nearer source of EMF causing higher signal strengths during active use. Our analysis on school EMF measurements was adjusting for mobile phone usage, which could also be encouraged for future studies.

Increasing SAR values of mobile phones were associated with increases in the prevalence of the symptoms headache, concentration difficulties, fatigue and sleep disturbances. We could not find a study using the SAR values of mobile phones participant were using. As it is a safety precaution issue and a regulated value, there might be other associations that we could not find, since these associations probably depend on usage as well. A student with a high-SAR phone might be using his phone less than another student with a low-SAR phone using it intensively.

The association we found between the position and status of the mobile phone at night with many general symptoms in our study is consistent with other study findings. It was reported that among adolescents; being awakened at night by mobile phone was associated with an increase in health symptom reports such as tiredness, rapid exhaustibility, headache and physical ill-being but not with memory and concentration capacity [43]. In a study on smaller children aged 9–12 years in Hungary, an association was found between going to bed later at night and regular mobile phone use for calls and text messaging [44]. In a one-year cohort study among college students, frequent mobile phone calls and text messaging was associated with difficulties falling asleep among male participants [37]. A Finnish survey on 7292 adolescents had found that information and communication technology use was linked to poor perceived health especially when it negatively acted on their sleeping habits, which in turn caused daytime tiredness and the link with intensive mobile phone usage was more pronounced for girls compared to computer predominance among boys [45].

Carrying the mobile phone on the clothes, in the pocket or bag was associated with increased headache, fatigue and sleep disturbance risks. There are some studies which showed that carrying a cell phone on a body was associated with harmful health risk due to the radiation. For men, a recent study has found an association between carrying the mobile phone switched on and erectile dysfunction [46] and for women, carrying a mobile phone in the bra has been linked with the development of breast cancer [47].

The students who kept their mobile phones switched on (either in normal or silent mode) while studying had significant increased risks of headache, fatigue, sleep disturbances and visual disturbances. Even when the mobile phones were on silent mode, they continue to exchange signals with base stations when they were open. These symptoms may be related with this low EMF. Recent studies showed that mobile phone and internet addiction has emerged as an important community health problem for adolescents [48–50]. Mobile phones were also indispensable for the adolescents’ lives; they perceived it as something they cannot live without [51]. Interaction with mobile phone while studying may also affect academic performance. Several studies have documented negative impact on academic performance [52, 53].

Making calls while charging was another exposure variable significantly associated with multiple general symptoms, namely headache, concentration difficulties, fatigue, sleep disturbances, visual disturbances and all of the local symptoms. While charging, there are two electrical currents, one to fill in the battery and the other to make the phone work, which cause a magnetic field in addition to the RF-EMF exposure.

All of the six local symptoms showed increasing trends with increasing number of calls per day and total duration of calls per day. Overall, it was consistent to find associations of local symptoms with call- and position- related independent variables, but not with variables like number of text messages, since all the local symptoms questioned were related to the head and sending text requires moving the phone away from the head. As such, we could also expect a protective effect of using earphones, which was absent for local symptoms but present for general symptoms in our data. A possible reason might the very few number of 17 students always using earphones in our study group. In the study among Swedish 15–19 year-olds, adjusting for use of hands-free equipment (*n* = 200) did not change the results on associations between symptoms and mobile phone use [33].

Warming of the ear was among the local symptoms showing an increasing trend with the number and duration of calls, which is supported by a human experimental study detecting increases in tympanic temperature after a continuous exposure of 60 min, but a decrease when exposure was intermittent, indicating also possible interactions with thermoregulatory mechanisms of the body [54]. The only local symptom associated with use of the firms’ promotions was again warming of the ear, and promotions could have an encouraging role on users for making more phone calls.

In our study, headache was the only local symptom associated with connection to the internet. A recent study has found significant changes in EEG activity after 15 min exposure to a 3G dialling mobile phone placed on the ear, but no significant change when the same phone was placed on the chest, indicating the importance of the position of the phone [7].

Among the local symptoms questioned, only flushing was associated with the position and status of mobile phone at night, although more associations were observed with general symptoms for this exposure. This may be related to the low intensity of the signal exchange with the base station of the mobile phone at night in the turned-on position. It may also be related to be held in a location more distant to the head.

Our study has several important limitations, the first being its cross-sectional nature limiting inferences. The questionnaire data rely on students’ self reports. The EMF measurements were conducted at schools; however students spend more time outside school. An important limitation of our EMF measurements is that they could not be taken from their classrooms and they were conducted 1 year after the questionnaires were applied (due to a delay in obtaining the devices from abroad) thus they might not represent the maximal points and values in 2010 due to a possibility of alteration or installation of base stations around schools. Besides, maximal values might not be representative of school EMF levels. Longer-duration measurements and calculating averages might be another approach, while maximal values are preferred in daily practice, to detect possible spots over exposure limits. The students’ preference to use their phones mostly for text messaging could be considered another limitation in the generalizibility of the results. The presence of non-users as a baseline control group is among the strengths of this study, in this era when it is hard to find people not using mobile phones. However, the disproportionately small number of 129 non-users as compared to 2021 users could be considered a limitation. Exploring dose-response with categorizations of exposure variables could be considered another advantage of this study. The limited number of 3G users, as it was recently introduced in Turkey during the data collection period, could be considered a limitation, to evaluate the impact of this new technology, however it could be important to have this data before 3G and the smart phone - Wi-Fi era since their frequency bands are different; 2100 and 2400, respectively, than the 2G bands at 900 and 1800 MHz and their health impacts might also be different.

## Conclusions

Our findings suggest an association between mobile phone use and some symptoms. Dose-response relationships were more conspicuous for the number of calls per day, total duration of calls per day, total number of text messages per day, position and status of mobile phone at night and making calls while charging as exposures and headache, concentration difficulties, fatigue and sleep disturbances as general symptoms and flushing and warming of the ear as local symptoms. We observed many statistically significant associations which cannot be expected by chance alone. While some of the observed associations showed a consistent pattern, some did not. We have found limited associations between vicinity to base stations and some general symptoms; however, we did not find any association with school EMF levels. Our school EMF measurements were very limited. We would recommend more specific school measurements like measuring from the desks of the students and also measuring EMF at home. Decreasing the number and duration of calls, decreasing messages, using earphones, keeping the phone away from the head and body and similar precautions might decrease the frequencies or prevalence of the symptoms.

## Additional files

Additional file 1: Questionnaire on risk perceptions, mobile phone use and related symptoms of high school students in Bornova. (DOC 104 kb)

Additional file 2: Presence of the remaining nine general symptoms with respect to mobile phone usage parameters; p trend, OR (adjusted for gender and school type) and 95% confidence intervals; non-users as the reference category of each analysis. (DOC 159 kb)

Additional file 3: Presence of general symptoms with respect to vicinity to base stations and school EMF levels; p trend, OR (adjusted for gender, school type and total duration of mobile phone calls per day classified into four categories as <5 min, 5-9 min, 10-30 min, >30 min) and 95% confidence intervals. (DOC 170 kb)

## Abbreviations

CI: Confidence interval
EMF: Electromagnetic field
OR: Odds ratio
RF: Radiofrequency
USD: United States dollars
