# Supplementary material for: Mobile phone use, school electromagnetic field levels and related symptoms: a cross-sectional survey among 2150 high school students in Izmir
Source: Environ Health. 2017 Jun 2;16:51. doi: 10.1186/s12940-017-0257-x (PMC5455117; doi:10.1186/s12940-017-0257-x)
Supplement: Supplementary file 1 — Questionnaire on risk perceptions, mobile phone use and related symptoms of high school students in Bornova. (DOC 104 kb) [file 12940_2017_257_MOESM1_ESM.doc]

**RISK PERCEPTIONS, MOBILE PHONE USE AND RELATED SYMPTOMS OF HIGH SCHOOL STUDENTS IN BORNOVA**

*Dear students,*

*A study is being conducted by the Public Health Department of the Ege University Medical School among high school students in Bornova in order to determine their mobile phone usage, perceptions on the possible risks of mobile phones and the frequency of some symptoms that may be related to mobile phone usage. We ask you to answer the questions in the following survey. You will also be given an education on this topic. Thank you for participating in the survey.*

***Socio-demographic characteristics***

1. School: …………………………………………………. High School

2. The program you are studying

1. Trade high school
2. Technical high school for girl
3. Standard high school
4. Industrial technical high school
5. Anatolian high school
6. Science high school
7. Private high school
8. Other *(please write clearly)……………..*

3. Your class and grade: ………………………

4. What is your date of birth? *(Specify day, month and year)* ……. / ……. / 19……

5. Your gender: 1. Male 2. Female

6. What do you think about the income level of your family?

1. Very good 2. Good 3. Moderate 4. Bad 5. Very bad

7. How much is your family’s total monthly income ……………… YTL

8. How many people live in your house? …………….. person

9. What is your father’s job?

1. Blue collar
2. Unemployed
3. Self Employed
4. Employer
5. White collar
6. Manager
7. Retired: *(please write the job before retirement)*………………………………..
8. Other: *(please write clearly)* …………….........................................................

10. Which school has your mother last finished?

1. Illiterate 4. Secondary
2. Only literate 5. High school
3. Primary 6. University

11. Which school has your father last finished?

1. Illiterate 4. Secondary

2. Only literate 5. High school

3. Primary 6. University

***Questions about mobile phone usage***

12. Do you use a mobile phone? 1. Yes 2. No

13. Do you have a mobile phone? 1. Yes 2. No

14. If yes; *(if you have more than one mobile phone, please state separately for each of them)*

1. The brand and model of your mobile phone: ………………..
2. The brand and model of your mobile phone: ………………..
3. The brand and model of your mobile phone: ………………..

15. What is the most important factor causing your parents to buy you a mobile phone?

1. My family’s will to be in touch with me
2. To communicate with friends more easily
3. Most of my friends’ ownership of mobile phones
4. Other *(please describe)*…………………………………………………

16. Since how many years have you been using a mobile phone? ……………… years

17. On the average, how many times a day do you speak on the phone?

………… times a day If you do not use it every day, …………. times a week

18. Do you know the SAR value of your mobile phone? 1. Yes: …………. 2. No

19. In total, approximately how many minutes per day do you speak with your mobile phone? ……………. minutes

20. Do you use earphones while speaking with your mobile phone?

1. Yes, always 2. Yes, frequently 3. Yes, sometimes 4. Yes, rarely

5. No, never

21. On the average, how many text messages do you send and receive per day? ………… messages

22. Do you connect to the internet via mobile phone? 1. Yes, ……… minutes per week 2. No

23. Please state the type(s) of your phone line’s (lines’) tariff plan.

1. Prepaid 2. Bill

24. If you use a prepaid tariff plan, on the average how much do you pay per month? ………

25. I you use a bill tariff plan, approximately how much is your monthly bill? ……… YTL

26. What is your tariff plan? …………………………..

27. Are you using any promotions or special offers? 1. Yes 2. No

28. If yes, please describe the promotion/ offer: …………………………………………………......

29. Is there a base station near your home or school?

1. Yes, there is one …...... meters from my home
2. Yes, there is one …...... meters from my school
3. No, there is none
4. I don’t know

30. While in bed at night, do you keep your mobile phone on your bedside?

1. Yes 2. No

31. If yes, do you keep it on or off?

1. Off 2. On

32. How many meters away do you keep it at night? ............... m

33. Where do you carry your mobile phone during daytime?

1. In the pocket of my daily clothes

2. In the pocket of my overcoat or jacket

3. On my belt

4. In my bag

5. Other *(Please describe)*……………………………………………………

34. In which state is your mobile phone while you are studying at a table?

1. Turned off

2. Turned on or in silent mode on myself

3. Turned on or in silent mode ………meters away

35. In which mode do you charge your mobile phone?

1. Turned off 2. Turned on

36. Do you make phone calls while charging your mobile phone?

1. Yes 2. No

***Questions on Risk Perception***

Please score the following questions with a circle between 1 and 5

1. What do you think about the risk of cell phones for public?

“1” no risk; “5” very high risk

1 2 3 4 5 🞏 Don’t know/No idea

1. What do you think about the benefit of cell phones for public?

“1” very beneficial; ‘5’ no benefit

1 2 3 4 5 🞏 Don’t know/No idea

1. Do you trust the institutions that control the mobile phones

“1” very high trust; ‘5’ no trust

1 2 3 4 5 🞏 Don’t know/No idea

1. What do you think about the the risk of base stations for public?

“1” no risk; “5” very high risk

1 2 3 4 5 🞏 Don’t know/No idea

1. The benefit of base stations for public?

“1” very beneficial; ‘5’ no benefit

1 2 3 4 5 🞏 Don’t know/No idea

1. Do you trust the institutions that control the base stations?

“1” very high trust; ‘5’ no trust

1 2 3 4 5 🞏 Don’t know/No idea

Please score the following statements with a circle between 1 and 5

‘1’ absolutely agree; ‘5’ no agreement.

| **Statement** | **Score** |
| --- | --- |
| 1. EMR pollution emitted by base stations is sufficiently regulated | 1 2 3 4 5 🞏 Don’t know |
| 1. The use of mobile phones should be limited to children and adolescents because of the potential health risks | 1 2 3 4 5 🞏 Don’t know |
| 1. People living close to a base station should be monitored | 1 2 3 4 5 🞏 Don’t know |
| 1. People living close to a base station should participate in the decision on where the antennas are built | 1 2 3 4 5 🞏 Don’t know |
| 1. Legal limit values should be determined considering the minimum value that may affect health | 1 2 3 4 5 🞏 Don’t know |
| 1. Electromagnetic radiation pollution from base stations causes cancer | 1 2 3 4 5 🞏 Don’t know |
| 1. Many of chemical substances cause cancer | 1 2 3 4 5 🞏 Don’t know |
| 1. The studies on the reactions of plants and animals expose to EMR would be a reliable predictor how humans would react | 1 2 3 4 5 🞏 Don’t know |
| 1. People are overly worried that they could develop a disease due to EMR pollution | 1 2 3 4 5 🞏 Don’t know |

What do you think about the risk of the following behaviors? Please score the following statements with a circle between 1 and 5. ‘1’ no risk; ‘5’ very high risk

| **Behavior** | **Score** |
| --- | --- |
| 1. To make a mobile phone call which takes 20 minutes | 1 2 3 4 5 🞏 Don’t know |
| 1. To carry a turned-on mobile phone during the whole day | 1 2 3 4 5 🞏 Don’t know |
| 1. To make a mobile phone call which takes an hour | 1 2 3 4 5 🞏 Don’t know |
| 1. To sleep all night with a turned-on mobile phone resting on the bed side table | 1 2 3 4 5 🞏 Don’t know |
| 1. To make a mobile phone call on the bus | 1 2 3 4 5 🞏 Don’t know |
| 1. Keeping your mobile phone open on the bus | 1 2 3 4 5 🞏 Don’t know |
| 1. To make a mobile phone call in the public area (Metro, cafe) | 1 2 3 4 5 🞏 Don’t know |

How rude do you think the following behaviors are? Please score the following statements with a circle between 1 and 5. ‘1’ no risk; ‘5’ very high risk

| **Behavior** | **Score** |
| --- | --- |
| 1. To leave a family lunch to answer the mobile phone | 1 2 3 4 5 🞏 Don’t know |
| 1. To have one’s mobile phone ring in a public area | 1 2 3 4 5 🞏 Don’t know |
| 1. To stop a conversation to answer the mobile phone | 1 2 3 4 5 🞏 Don’t know |

***Questions on symptoms that might be related to mobile phone use***

Have you ever had any of the following symptoms in the past one month?

Please circle by giving a score between 1 and 5. “1” means never, “5” means very frequent.

| **Symptom experienced in the past one month** | **Frequency**  “1” means never, “5” means very frequent. |
| --- | --- |
| 1. Headache | 1 2 3 4 5 |
| 1. Dizziness | 1 2 3 4 5 |
| 1. Concentration difficulties | 1 2 3 4 5 |
| 1. Forgetfulness | 1 2 3 4 5 |
| 1. Fatigue | 1 2 3 4 5 |
| 1. Sleep disturbances | 1 2 3 4 5 |
| 1. Blurred vision/ visual disturbances | 1 2 3 4 5 |
| 1. Tremor | 1 2 3 4 5 |
| 1. Depressive symptoms (sensation of sadness) | 1 2 3 4 5 |
| 1. Nervousness, irritability | 1 2 3 4 5 |
| 1. Nausea | 1 2 3 4 5 |
| 1. Loss of appetite | 1 2 3 4 5 |
| 1. Palpitation, change in cardiac rhythm | 1 2 3 4 5 |
| 1. Dryness in the throat | 1 2 3 4 5 |
| 1. Sensitivity towards sounds | 1 2 3 4 5 |
| 1. Difficulties in hearing | 1 2 3 4 5 |
| 1. Allergy | 1 2 3 4 5 |

While you are having a call with mobile phone, do you experience any of the following symptoms on the side you use the mobile phone?

Please circle by giving a score between 1 and 5. “1” means never, “5” means very frequent.

| **Symptom** | **Frequency** “1” means never, “5” means very frequent. |
| --- | --- |
| 1. Feeling of discomfort | 1 2 3 4 5 |
| 1. Flushing in the face | 1 2 3 4 5 |
| 1. Prickling in the ear | 1 2 3 4 5 |
| 1. Warming of the ear | 1 2 3 4 5 |
| 1. Headache | 1 2 3 4 5 |
| 1. Numbness in the head or face | 1 2 3 4 5 |

*Thank you for responding thoroughly.*

*Hope to see you in the educational session ☺*
