# Supplementary material for: Mobile phone use, school electromagnetic field levels and related symptoms: a cross-sectional survey among 2150 high school students in Izmir
Source: Environ Health. 2017 Jun 2;16:51. doi: 10.1186/s12940-017-0257-x (PMC5455117; doi:10.1186/s12940-017-0257-x)
Supplement: Supplementary file 2 — Presence of the remaining nine general symptoms with respect to mobile phone usage parameters; p trend, OR (adjusted for gender and school type) and 95% confidence intervals; non-users as the reference category of each analysis. (DOC 159 kb) [file 12940_2017_257_MOESM2_ESM.doc]

Additional file 1. Presence of the remaining nine general symptoms with respect to mobile phone usage parameters; p trend, OR (adjusted for gender and school type) and 95% confidence intervals; non-users as the reference category of each analysis

| Characteristic | Forgetfulness | | | Tremor | | Irritability | | Nausea | | Loss of appetite | | Arrythmia | | Sensitivity towards sounds | | Hearing difficulties | | Allergy | |
| --- | --- | --- | --- | --- | --- | --- | --- | --- | --- | --- | --- | --- | --- | --- | --- | --- | --- | --- | --- |
|  | n (%) | | p trend/ OR | n (%) | p trend/ OR | n (%) | p trend/ OR | n (%) | p trend/ OR | n (%) | p trend/ OR | n (%) | p trend/ OR | n (%) | p trend/ OR | n (%) | p trend/ OR | n (%) | p trend/ OR |
| Mobile phone use | | | 0.055* |  | 0.688* |  | **0.049*** |  | 0.876* |  | 0.805* |  | 0.655* |  | 0.337* |  | 0.114* |  | 0.300* |
| Non-users | 66  (52.4) | | 1 | 25 (19.8) | 1 | 72 (57.6) | 1 | 30 (23.8) | 1 | 38 (30.9) |  | 37  (29.8) | 1 | 47 (38.2) | 1 | 41 (33.3) | 1 | 21 (16.8) | 1 |
| Current users | 1219 (61.0) | | 1.18  (0.81-1.72) | 427 (21.4) | 1.15  (0.73-1.82) | 1331 (66.2) | 1.22  (0.83-1.77) | 488 (24.4) | 0.93  (0.60-1.43) | 593 (29.8) | 0.93  (0.62-1.38) | 633 (31.7) | 1.10  (0.73-1.64) | 849 (42.6) | 1.05  (0.72-1.54) | 537 (26.8) | 0.71  (0.48-1.05) | 271 (13.5) | 0.77  (0.47-1.26) |
| Number of calls per day |  | | 0.128 |  | 0.095 |  | **0.010** |  | 0.573 |  | 0.281 |  | 0.099 |  | 0.162 |  | 0.660 |  | 0.715 |
| <1 call | 294  (59.9) | | 1.13  (0.75-1.70) | 101 (20.5) | 1.08  (0.66-1.78) | 318 (64.4) | 1.11  (0.73-1.68) | 122 (24.8) | 0.92  (0.58-1.48) | 149 (30.4) | 0.93  (0.60-1.44) | 144 (29.3) | 0.96  (0.62-1.49) | 207 (42.0) | 0.99  (0.65-1.49) | 128 (26.0) | 0.68  (0.44-1.05) | 65 (13.2) | 0.74  (0.43-1.28) |
| 1-4 calls | 681  (62.4) | | 1.24  (0.84-1.82) | 223 (20.5) | 1.07  (0.67-1.71) | 731 (66.6) | 1.22  (0.83-1.80) | 250 (22.9) | 0.83  (0.53-1.29) | 299 (27.6) | 0.81  (0.54-1.23) | 342 (31.5) | 1.08  (0.72-1.64) | 459 (42.3) | 1.02  (0.69-1.51) | 277 (25.3) | 0.65  (0.43-0.97) | 136 (12.4) | 0.69  (0.41-1.15) |
| ≥5 calls | 190  (60.7) | | 1.26  (0.82-1.94) | 83 (26.4) | 1.58  (0.94-2.63) | 220 (70.3) | **1.58**  **(1.02-2.46)** | 86 (27.7) | 1.15  (0.70-1.88) | 115 (37.0) | 1.30  (0.82-2.04) | 110 (35.3) | 1.34***  (0.85-2.12) | 143 (46.0) | 1.25  (0.80-1.93) | 101 (32.5) | 0.94  (0.60-1.48) | 53 (16.9) | 0.94  (0.53-1.65) |
| Total duration of calls per day | | | **0.001** |  | 0.061 |  | **<0.001** |  | **0.017** |  | 0.057 |  | **0.001** |  | **<0.001** |  | 0.868 |  | 0.376 |
| <5 minutes | 336  (58.4) | | 1.16  (0.77-1.73) | 123 (21.3) | 1.19  (0.73-1.94) | 350 (60.4) | 0.99  (0.66-1.48) | 124 (21.5) | 0.82  (0.52-1.31) | 165 (28.6) | 0.90  (0.58-1.38) | 165 (28.5) | 0.97  (0.63-1.50) | 228 (39.5) | 0.94  (0.62-1.42) | 154 (26.6) | 0.72  (0.47-1.10) | 74 (12.8) | 0.74  (0.43-1.27) |
| 5-9 min. | 196  (57.1) | | 1.06  (0.70-1.63) | 59 (17.3) | 0.91  (0.54-1.54) | 218 (63.7) | 1.13  (0.74-1.74) | 81 (23.6) | 0.93  (0.57-1.52) | 92 (27.1) | 0.82  (0.52-1.30) | 98  (28.5) | 0.97  (0.61-1.53) | 140 (40.7) | 0.98  (0.64-1.52) | 88 (25.6) | 0.67  (0.43-1.06) | 42 (12.2) | 0.68  (0.38-1.21) |
| 10-30 min. | 450  (63.6) | | 1.26  (0.85-1.88) | 158 (22.3) | 1.20  (0.74-1.94) | 496 (69.7) | 1.38  (0.93-2.06) | 180 (25.5) | 0.97  (0.61-1.52) | 210 (30.0) | 0.92  (0.60-1.41) | 231 (32.9) | 1.56  (0.76-1.77) | 312 (44.4) | 1.12  (0.75-1.68) | 192 (27.2) | 0.72  (0.47-1.10) | 106 (14.9) | 0.88  (0.52-1.50) |
| >30 min. | 154  (66.7) | | 1.34  (0.85-2.12) | 64 (27.8) | 1.54  (0.91-2.64) | 176 (75.5) | **1.78**  **(1.11-2.87)** | 70 (30.2) | 1.19  (0.72-1.98) | 89 (38.7) | 1.32  (0.82-2.13) | 98 (42.6) | **1.68**  **(1.05-2.70)** | 126 (54.8) | 1.66  (1.05-2.62) | 72 (30.9) | 0.85  (0.53-1.37) | 37 (16.0) | 0.89  (0.49-1.63) |
| Use of earphones during calls | | | 0.076 |  | 0.491 |  | **0.016** |  | 0.808 |  | 0.550 |  | 0.995 |  | 0.451 |  | 0.116 |  | 0.071 |
| Always | 8  (47.1) | | 0.60  (0.21-1.71) | 4  (23.5) | 1.33  (0.39-4.47) | 10 (58.8) | 0.78  (0.27-2.25) | 6  (37.5) | 1.59  (0.53-4.81) | 7  (41.2) | 1.49  (0.52-4.26) | 6  (37.5) | 1.40  (0.47-4.18) | 8  (47.1) | 1.13  (0.40-3.18) | 6  (35.3) | 1.05  (0.36-3.08) | 3  (17.6) | 0.97  (0.25-3.76) |
| Often/ sometimes | 212  (62.2) | | 1.20  (0.78-1.84) | 69 (20.1) | 1.01  (0.60-1.69) | 220 (64.0) | 1.06  (0.69-1.63) | 85 (24.8) | 0.91  (0.56-1.48) | 105 (30.8) | 0.94  (0.60-1.48) | 114 (33.4) | 1.16  (0.74-1.82) | 147 (43.0) | 1.04  (0.67-1.60) | 93 (27.1) | 0.70  (0.45-1.10) | 55 (16.0) | 0.87  (0.50-1.54) |
| Rarely/ never | 978  (61.2) | | 1.21  (0.83-1.77) | 349 (21.8) | 1.20  (0.76-1.90) | 1079 (67.1) | 1.27  (0.87-1.86) | 386 (24.2) | 0.93  (0.60-1.43) | 471 (29.7) | 0.93  (0.62-1.40) | 504 (31.6) | 1.10  (0.73-1.65) | 681 (42.8) | 1.06  (0.72-1.57) | 428 (26.7) | 0.72  (0.48-1.07) | 204 (12.7) | 0.72  (0.44-1.19) |
| Total no.of texting per day | | | **0.041** |  | **0.043** |  | **0.002** |  | 0.188 |  | **0.037** |  | **0.020** |  | 0.133 |  | 0.347 |  | 0.572 |
| No text message | 29  (50.0) | | 1.01  (0.53-1.91) | 13 (22.0) | 1.28  (0.60-2.76) | 29 (49.2) | 0.74  (0.39-1.40) | 13 (22.0) | 0.99  (0.47-2.10) | 10 (17.2) | 0.47  (0.21-1.04) | 16  (27.1) | 0.94  (0.47-1.90) | 23 (39.0) | 1.05  (0.55-2.01) | 16 (27.1) | 0.77  (0.39-1.54) | 6 (10.2) | 0.58  (0.22-1.54) |
| <15 | 241  (62.4) | | 1.41  (0.92-2.17) | 68 (17.8) | 0.93  (0.55-1.59) | 244 (63.7) | 1.14  (0.74-1.75) | 93 (24.3) | 0.98  (0.60-1.61) | 108 (28.3) | 0.86  (0.55-1.37) | 115 (30.1) | 1.04  (0.66-1.64) | 160 (41.8) | 1.05  (0.68-1.62) | 104 (27.1) | 0.73  (0.46-1.15) | 46 (11.9) | 0.68  (0.38-1.22) |
| 15-74 | 265  (59.2) | | 1.06  (0.70-1.62) | 92 (20.3) | 1.11  (0.67-1.84) | 309 (68.1) | 1.26  (0.83-1.93) | 101 (22.5) | 0.82  (0.50-1.32) | 130 (28.9) | 0.88  (0.56-1.37) | 125 (27.9) | 0.89  (0.57-1.40) | 190 (42.6) | 1.03  (0.67-1.57) | 121 (26.8) | 0.71  (0.46-1.11) | 52 (11.6) | 0.67  (0.38-1.19) |
| 75-199 | 273  (63.5) | | 1.25  (0.82-1.90) | 96 (22.5) | 1.19  (0.72-1.96) | 296 (68.5) | 1.29  (0.85-1.97) | 102 (23.8) | 0.84  (0.52-1.35) | 122 (28.4) | 0.83  (0.54-1.30) | 132 (30.8) | 0.99  (0.64-1.55) | 185 (43.0) | 1.03  (0.68-1.57) | 113 (26.3) | 0.68  (0.44-1.05) | 68 (15.7) | 0.93  (0.54-1.60) |
| ≥200 text messages | 337  (62.2) | | 1.22  (0.82-1.83) | 131 (24.1) | 1.25  (0.77-2.04) | 373 (68.3) | 1.33  (0.89-2.00) | 150 (27.6) | 1.07  (0.68-1.70) | 186 (34.7) | 1.13  (0.74-1.73) | 198 (36.6) | 1.30  (0.85-2.00) | 242 (44.7) | 1.15  (0.76-1.72) | 148 (27.2) | 0.71  (0.46-1.08) | 76 (14.0) | 0.74  (0.43-1.27) |
| Use of promotions | | | **0.002** |  | 0.958 |  | **<0.001** |  | 0.777 |  | 0.734 |  | 0.190 |  | **0.016** |  | 0.233 |  | 0.895 |
| No | 308  (56.9) | | 1.07  (0.72-1.60) | 119 (21.8) | 1.18  (0.72-1.92) | 332 (61.1) | 1.05  (0.70-1.57) | 130 (24.0) | 0.94  (0.59-1.49) | 158 (29.0) | 0.91  (0.59-1.39) | 161 (29.7) | 1.02  (0.66-1.57) | 207 (38.2) | 0.92  (0.61-1.38) | 148 (27.1) | 0.72  (0.47-1.11) | 66 (12.1) | 0.69  (0.40-1.18) |
| Yes | 881  (62.9) | | 1.25  (0.85-1.82) | 293 (21) | 1.12  (0.70-1.78) | 970 (68.8) | 1.34  (0.91-1.97) | 343 (24.5) | 0.91  (0.59-1.42) | 422 (30.5) | 0.95  (0.63-1.43) | 458 (32.9) | 1.14  (0.75-1.71) | 616 (44.3) | 1.10  (0.74-1.62) | 374 (26.7) | 0.70  (0.47-1.04) | 197 (14.1) | 0.80  (0.48-1.33) |
| SAR value of mobile phone | | | 0.137 |  | 0.526 |  | 0.054 |  | 0.394 |  | 0.841 |  | 0.696 |  | 0.714 |  | 0.080 |  | 0.525 |
| <0.50 | 97  (59.1) | | 1.05  (0.65-1.71) | 30 (18.5) | 0.95  (0.52-1.72) | 105 (64.0) | 1.06  (0.65-1.73) | 34 (20.9) | 0.74  (0.42-1.31) | 46 (28.2) | 0.85  (0.50-1.42) | 47  (28.8) | 0.91  (0.54-1.53) | 75 (46.0) | 1.17  (0.72-1.90) | 45 (27.4) | 0.72  (0.43-1.20) | 21 (12.9) | 0.74  (0.38-1.45) |
| 0.50-<0.75 | 428  (62.3) | | 1.22  (0.82-1.82) | 141 (20.5) | 1.07  (0.66-1.73) | 462 (66.6) | 1.21  (0.81-1.81) | 172 (25.1) | 0.97  (0.62-1.53) | 208 (30.5) | 0.94  (0.61-1.43) | 221 (32.1) | 1.08  (0.71-1.65) | 287 (41.8) | 0.99  (0.66-1.49) | 186 (27.0) | 0.70  (0.46-1.06) | 91 (13.2) | 0.74  (0.43-1.25) |
| ≥0.75 | 431  (61.1) | | 1.17  (0.79-1.75) | 149 (21.1) | 1.17  (0.72-1.90) | 476 (67.2) | 1.126  (0.84-1.89) | 180 (25.4) | 1.01  (0.64-1.60) | 206 (29.3) | 0.91  (0.59-1.39) | 219 (31.1) | 1.06  (0.69-1.63) | 300 (42.7) | 1.04  (0.69-1.57) | 179 (25.2) | **0.64**  **(0.42-0.98)** | 95 (13.4) | 0.80  (0.47-1.37) |
| Position and status of mobile phone at night | | | **<0.001** |  | 0.051 |  | 0.085 |  | 0.086 |  | 0.134 |  | 0.551 |  | 0.212 |  | 0.724 |  | 0.915 |
| Off | 168  (56.8) | | 1.06  (0.68-1.66) | 65 (21.7) | 1.20  (0.69-2.07) | 205 (68.1) | 1.45  (0.92-2.29) | 61 (20.5) | 0.86  (0.50-1.45) | 82 (27.6) | 0.84  (0.52-1.35) | 93  (31.4) | 1.02  (0.64-1.65) | 131 (44.0) | 1.09  (0.69-1.72) | 76 (25.5) | 0.66  (0.41-1.08) | 38 (12.8) | 0.75  (0.40-1.40) |
| On, ≥1 m away | 320  (59.6) | | 1.19  (0.78-1.80) | 111 (20.7) | 1.11  (0.67-1.85) | 360 (66.8) | 1.34  (0.88-2.03) | 139 (25.8) | 1.1  (0.68-1.78) | 160 (30.0) | 0.93  (0.60-1.45) | 181  (33.7) | 1.12  (0.72-1.74) | 241 (44.7) | 1.14  (0.75-1.74) | 149 (27.6) | 0.74  (0.48-1.15) | 80 (14.8) | 0.90  (0.52-1.58) |
| On, 25-99cm away | 178  (68.7) | | **1.76**  **(1.11-2.79)** | 58 (22.5) | 1.22  (0.70-2.11) | 169 (65.0) | 1.21  (0.76-1.91) | 63 (24.6) | 1.02  (0.61-1.73) | 76 (29.7) | 0.91  (0.56-1.47) | 79  (30.7) | 0.96  (0.59-1.55) | 103 (40.1) | 0.92  (0.58-1.47) | 77 (29.7) | 0.82  (0.51-1.32) | 34 (13.2) | 0.74  (0.40-1.38) |
| On, 0-24 cm away | 133  (65.8) | | 1.55  (0.96-2.5) | 58 (28.7) | 1.65  (0.95-2.88) | 145 (71.4) | 1.61  (0.99-2.63) | 57 (28.4) | 1.25  (0.73-2.14) | 73 (36.7) | 1.23  (0.75-2.02) | 70  (35.0) | 1.17  (0.71-1.92) | 99 (49.7) | 1.37  (0.85-2.21) | 60 (29.7) | 0.8  (0.48-1.31) | 31 (15.3) | 0.82  (0.44-1.56) |
| Phone position during daytime | | | 0.014 |  | 0.388 |  | **0.023** |  | 0.278 |  | 0.759 |  | 0.138 |  | 0.641 |  | 0.229 |  | 0.123 |
| Does not carry; leaves at home or in a furniture at school | 42  (49.4) | | 0.83  (0.47-1.47) | 11 (13.3) | 0.65  (0.30-1.43) | 50 (59.5) | 1.02  (0.57-1.82) | 15 (18.1) | 0.75  (0.36-1.53) | 19 (23.5) | 0.67  (0.35-1.29) | 19  (22.6) | 0.74  (0.38-1.42) | 39 (46.4) | 1.26  (0.71-2.25) | 23 (27.4) | 0.82  (0.44-1.53) | 13 (15.5) | 0.87  (0.40-1.87) |
| On his clothes/ in his pocket/ bag | 1051 (61.5) | | 1.20  (0.81-1.77) | 364 (21.2) | 1.13  (0.71-1.81) | 1146 (66.6) | 1.26  (0.85-1.86) | 421 (24.6) | 1.06  (0.67-1.68) | 517 (30.3) | 0.96  (0.63-1.45) | 550  (32.2) | 1.22  (0.80-1.87) | 720 (42.2) | 1.01  (0.68-1.50) | 456 (26.6) | 0.74  (0.49-1.12) | 225 (13.1) | 0.68  (0.41-1.13) |
| Status of phone while studying | | | **0.030** |  | 0.316 |  | **0.007** |  | 0.507 |  | 0.706 |  | 0.285 |  | 0.413 |  | 0.083 |  | 0.086 |
| Off | 91  (57.6) | | 1.08  (0.66-1.77) | 27 (17.3) | 0.96  (0.51-1.78) | 95 (60.9) | 1.03  (0.62-1.69) | 35 (22.4) | 0.92  (0.51-1.64) | 40 (26.1) | 0.79  (0.46-1.35) | 45  (28.8) | 1.00  (0.59-1.71) | 68 (43.6) | 1.10  (0.67-1.81) | 48 (30.6) | 0.91  (0.54-1.53) | 25 (15.9) | 0.88  (0.46-1.69) |
| On/ silent mode | 964  (61.7) | | 1.21  (0.82-1.78) | 334 (21.3) | 1.22  (0.76-1.97) | 1062 (67.4) | 1.33  (0.90-1.97) | 383 (24.5) | 1.01  (0.64-1.60) | 472 (30.3) | 0.98  (0.65-1.48) | 504 (32.3) | 1.20  (0.79-1.83) | 670 (43.0) | 1.06  (0.72-1.58) | 414 (26.4) | 0.72  (0.48-1.09) | 203 (12.9) | 0.68  (0.41-1.13) |
| Makes calls while charging | | | **<0.001** |  | 0.176 |  | **0.001** |  | **0.001** |  | 0.058 |  | **0.006** |  | **0.005** |  | 0.054 |  | **0.019** |
| No | 490  (56.6) | | 1.01  (0.66-1.55) | 165 (19.0) | 0.95  (0.57-1.57) | 547 (62.7) | 1.06  (0.69-1.62) | 178 (20.5) | 0.91  (0.55-1.51) | 231 (26.8) | 0.76  (0.49-1.19) | 245 (28.3) | 0.98  (0.62-1.55) | 339 (39.1) | 0.92  (0.60-1.42) | 199 (22.8) | **0.57**  **(0.36-0.89)** | 89 (10.2) | 0.55  (0.31-0.97) |
| Yes | | 615  (64.6) | 1.33  (0.87-2.02) | 212 (22.3) | 1.12  (0.68-1.85) | 663 (69.2) | 1.33  (0.87-2.03) | 264 (27.7) | 1.31  (0.79-2.16) | 310 (32.7) | 0.99  (0.63-1.54) | 330 (34.8) | 1.29  (0.82-2.02) | 431 (45.6) | 1.18  (0.77-1.81) | 285 (30.0) | 0.81  (0.52-1.26) | 154 (16.2) | 0.90  (0.52-1.55) |

**Bold, black ORs:** Significant ORs

**Bold, red p:** Significant p trends

*Pearson Chi-square (not p for trend due to number or quality of categories to compare

**p=0.051

***OR is **1.81 (1.02-3.20)** for the category ≥10 calls/day and 1.17 (0.72-1.90) for the category 5-9 calls/day when these two categories are separated.
