# Supplementary material for: Mobile phone use, school electromagnetic field levels and related symptoms: a cross-sectional survey among 2150 high school students in Izmir
Source: Environ Health. 2017 Jun 2;16:51. doi: 10.1186/s12940-017-0257-x (PMC5455117; doi:10.1186/s12940-017-0257-x)
Supplement: Supplementary file 3 — Presence of general symptoms with respect to vicinity to base stations and school EMF levels; p trend, OR (adjusted for gender, school type and total duration of mobile phone calls per day classified into four categories as <5 min, 5-9 min, 10-30 min, >30 min) and 95% confidence intervals. (DOC 170 kb) [file 12940_2017_257_MOESM3_ESM.doc]

Additional file 2. Presence of general symptoms with respect to vicinity to base stations and school EMF levels; p trend, OR (adjusted for gender, school type and total duration of mobile phone calls per day classified into four categories as <5 min, 5-9 min., 10-30 min., >30 min.) and 95% confidence intervals.

| Presence and location of base stations and school RF measurements | Headache | Dizziness | Concentration difficulties | Forgetfulness | Fatigue | Sleep disturbances | Visual disturbances | Tremor | Depressive symptoms | Irritability | Nausea | Loss of appetite | Arrythmia | Dryness of the throat | Sensitivity towards sounds | Difficulties in hearing | Allergy |  |
| --- | --- | --- | --- | --- | --- | --- | --- | --- | --- | --- | --- | --- | --- | --- | --- | --- | --- | --- |
| Base station near home/school (n=924)* | 0.151** | 0.337** | **0.014**** | **0.021**** | 0.449** | 0.542** | 0.130** | 0.923** | 0.170** | 0.184** | 0.929** | 0.148** | **0.024**** | 0.087** | **0.033**** | **0.016**** | 0.326** |  |
| No | 1 | 1 | 1 | 1 | 1 | 1 | 1 | 1 | 1 | 1 | 1 | 1 | 1 | 1 | 1 | 1 | 1 |  |
| Yes, near home | 1.38  (0.96-1.97) | 1.34  (0.97-1.87) | **1.52**  **(1.08-2.14)** | **1.48**  **(1.06-2.07)** | 1.13  (0.77-1.65) | 1.13  (0.82-1.57) | **1.44**  **(1.03-1.99)** | 1.11  (0.75-1.64) | 1.31  (0.95-1.82) | 1.21  (0.86-1.71) | 1.08  (0.74-1.58) | 1.28  (0.90-1.81) | **1.44**  **(1.02-2.03)** | 1.35  (0.97-1.88) | **1.44**  **(1.04-2.00)** | **1.58**  **(1.10-2.28)** | 1.20  (0.76-1.89) | |
| Yes, near school | 1.23  (0.78-1.92) | 1.17  (0.77-1.77) | 0.88  (0.57-1.33) | 1.32  (0.86-2.02) | 1.02  (0.63-1.66) | 1.17  (0.77-1.78) | 1.30  (0.86-1.99) | 1.02  (0.62-1.69) | 1.05  (0.69-1.6) | 0.94  (0.61-1.44) | 1.17  (0.73-1.89) | 0.91  (0.57-1.45) | 0.75  (0.46-1.20) | 1.03  (0.66-1.58) | 1.11  (0.72-1.69) | 0.90  (0.55-1.49) | 0.75  (0.39-1.44) | |
| Yes, near both home and school | 1.33  (0.76-2.34) | 1.01  (0.59-1.73) | 1.18  (0.69-2.01) | 0.88  (0.52-1.49) | 1.20  (0.65-2.2) | 1.25  (0.74-2.12) | 1.23  (0.73-2.07) | 1.03  (0.54-1.93) | 0.98  (0.58-1.65) | 1.43  (0.82-2.48) | 1.00  (0.54-1.86) | 1.33  (0.77-2.28) | 1.34  (0.78-2.31) | 0.79  (0.45-1.39) | **1.76**  **(1.05-2.96)** | **2.03**  **(1.18-3.5)** | 1.48  (0.75-2.91) | |
| Distance to base station (n=924)* | 0.125** | 0.359** | 0.361** | 0.124** | 0.472** | 0.056** | **0.001**** | 0.151** | 0.700** | 0.492** | 0.206** | 0.167** | **0.043**** | 0.197** | **0.007**** | **0.006**** | 0.351** |  |
| None or >300 meter away | 1 | 1 | 1 | 1 | 1 | 1 | 1 | 1 | 1 | 1 | 1 | 1 | 1 | 1 | 1 | 1 | 1 |  |
| ≤300 meter away | 1.18  (0.87-1.61) | 1.18  (0.89-1.57) | 1.09  (0.81-1.45) | 1.30  (0.97-1.74) | 1.00  (0.72-1.39) | 1.25  (0.94-1.66) | **1.56**  **(1.17-2.07)** | 1.27  (0.90-1.79) | 1.01  (0.76-1.34) | 1.05  (0.78-1.41) | 1.22  (0.88-1.70) | 1.24  (0.92-1.68) | **1.37**  **(1.01-1.85)** | 1.23  (0.92-1.65) | **1.43**  **(1.08-1.91)** | **1.76**  **(1.28-2.42)** | 1.31  (0.88-1.95) |  |
| School building RF | 0.304 | 0.696 | **<0.001** | 0.211 | **0.009** | 0.114 | 0.763 | 0.771 | **0.019** | 0.059 | 0.683 | 0.655 | 0.331 | 0.647 | 0.221 | 0.344 | 0.508 |  |
| 1^st^ quartile (≤0.602 V/m) | 1 | 1 | 1 | 1 | 1 | 1 | 1 | 1 | 1 | 1 | 1 | 1 | 1 | 1 | 1 | 1 | 1 |  |
| 2^nd^ quartile (0.603-0.850 V/m) | 0.87  (0.65-1.16) | 0.74  (0.56-0.96) | 0.81  (0.62-1.07) | 0.96  (0.74-1.26) | 1.01  (0.73-1.39) | 0.87  (0.67-1.13) | 0.90  (0.69-1.18) | 1.11  (0.81-1.53) | 0.83  (0.64-1.09) | 0.90  (0.68-1.18) | 1.12  (0.83-1.51) | 1.12  (0.85-1.49) | 1.19  (0.90-1.58) | 1.15  (0.87-1.5) | 1.24  (0.95-1.61) | 1.26  (0.94-1.69) | 1.06  (0.72-1.56) |  |
| 3^rd^ quartile (0.851-1.51 V/m) | 0.90  (0.65-1.24) | 1.13  (0.85-1.51) | 0.77  (0.57-1.05) | 1.09  (0.81-1.47) | 0.95  (0.67-1.34) | 0.77  (0.58-1.04) | 0.92  (0.69-1.23) | 1.25  (0.88-1.76) | 0.86  (0.64-1.16) | 0.79  (0.58-1.07) | **1.39**  **(1.00-1.92)** | 0.99  (0.72-1.35) | 1.2  (0.89-1.63) | **1.45**  **(1.08-1.95)** | **1.50**  **(1.12-2.00)** | **1.50**  **(1.09-2.06)** | **1.54**  **(1.02-2.32)** |  |
| 4^th^ quartile (≥1.52 V/m) | 0.94  (0.68-1.31) | 0.91  (0.68-1.21) | 0.81  (0.60-1.10) | 0.80  (0.60-1.08) | 0.78  (0.55-1.10) | 0.81  (0.60-1.08) | 1.06  (0.79-1.41) | 0.79  (0.55-1.13) | 0.77  (0.58-1.03) | 0.97  (0.71-1.32) | 1.07  (0.77-1.50) | 0.99  (0.73-1.36) | 1.16  (0.85-1.58) | 0.92  (0.68-1.24) | 1.00  (0.74-1.33) | 1.13  (0.82-1.56) | 0.83  (0.55-1.25) |  |
| School building 3G | **0.006** | 0.283 | **<0.001** | 0.118 | **<0.001** | 0.066 | 0.297 | 0.736 | **0.008** | **0.009** | 0.209 | 0.198 | 0.577 | 0.880 | 0.477 | 0.475 | **0.018** |  |
| 1^st^ quartile (≤0.0107 V/m) | 1 | 1 | 1 | 1 | 1 | 1 | 1 | 1 | 1 | 1 | 1 | 1 | 1 | 1 | 1 | 1 | 1 |  |
| 2^nd^ quartile (0.0108-0.024 V/m) | 1.00  (0.72-1.39) | 1.05  (0.78-1.40) | 0.76  (0.56-1.03) | 1.09  (0.81-1.47) | 1.00  (0.69-1.44) | 0.82  (0.62-1.10) | 1.02  (0.76-1.36) | 0.81  (0.57-1.16) | 0.85  (0.64-1.14) | 0.82  (0.60-1.11) | 1.07  (0.78-1.49) | 0.95  (0.69-1.29) | 0.96  (0.71-1.31) | 1.34  (1.00-1.79) | **1.47**  **(1.10-1.96)** | **1.38**  **(1.00-1.89)** | **1.76**  **(1.14-2.71)** |  |
| 3^rd^ quartile (0.025-0.0954 V/m) | 1.03  (0.69-1.54) | 0.98  (0.69-1.40) | 1.04  (0.71-1.51) | 1.09  (0.76-1.57) | 0.67  (0.44-1.04) | **0.70**  **(0.49-1.00)** | 0.90  (0.63-1.28) | 0.82  (0.53-1.26) | 0.91  (0.64-1.30) | 0.78  (0.54-1.14) | 1.30  (0.88-1.91) | 0.97  (0.67-1.42) | 1.16  (0.80-1.67) | 1.11  (0.78-1.60) | 1.14  (0.80-1.61) | 1.02  (0.69-1.52) | 1.03  (0.60-1.78) |  |
| 4^th^ quartile (≥0.0955 V/m) | 1.16  (0.74-1.83) | 1.05  (0.70-1.57) | 0.98  (0.64-1.49) | 1.15  (0.76-1.74) | 0.82  (0.51-1.32) | 0.72  (0.48-1.09) | 1.18  (0.79-1.76) | 1.25  (0.78-1.98) | 1.02  (0.68-1.53) | 1.05  (0.68-1.61) | 1.23  (0.79-1.92) | 0.92  (0.60-1.42) | 1.16  (0.77-1.76) | **1.58**  **(1.04-2.40)** | **1.51**  **(1.01-2.25)** | 1.49  (0.96-2.33) | **2.16**  **(1.23-3.81)** |  |
| School building 900 MHz | 0.444 | 0.459 | **0.004** | 0.824 | 0.640 | 0.860 | 0.429 | 0.311 | 0.185 | 0.208 | 0.503 | 0.741 | 0.563 | 0.736 | 0.336 | 0.373 | 0.673 |  |
| 1^st^ quartile (≤0.0602 V/m) | 1 | 1 | 1 | 1 | 1 | 1 | 1 | 1 | 1 | 1 | 1 | 1 | 1 | 1 | 1 | 1 | 1 |  |
| 2^nd^ quartile (0.0603-0.24 V/m) | 1.00  (0.75-1.35) | 0.82  (0.63-1.07) | 0.82  (0.62-1.08) | 1.13  (0.86-1.48) | 1.09  (0.79-1.49) | 0.92  (0.71-1.21) | 0.86  (0.66-1.12) | 1.06  (0.76-1.48) | 1.04  (0.79-1.36) | 0.89  (0.67-1.18) | 0.98  (0.72-1.33) | 1.01  (0.76-1.35) | 0.94  (0.71-1.25) | **1.41**  **(1.07-1.86)** | **1.33**  **(1.01-1.74)** | 1.26  (0.94-1.7) | 1.36  (0.90-2.05) |  |
| 3^rd^ quartile (0.25-0.478 V/m) | 0.75  (0.55-1.01) | 0.92  (0.70-1.20) | 0.85  (0.64-1.12) | 1.17  (0.89-1.55) | 0.96  (0.69-1.32) | 0.90  (0.69-1.19) | 0.79  (0.61-1.04) | **1.47**  **(1.07-2.01)** | 0.83  (0.63-1.09) | 0.77  (0.58-1.02) | 1.16  (0.86-1.57) | 0.98  (0.74-1.31) | 1.02  (0.77-1.35) | 1.26  (0.95-1.66) | **1.49**  **(1.14-1.95)** | 1.32  (0.98-1.77) | 1.27  (0.86-1.86) |  |
| 4^th^ quartile (≥0.479 V/m) | 0.75  (0.50-1.13) | 0.75  (0.53-1.06) | 0.77  (0.53-1.12) | 0.82  (0.57-1.18) | 0.69  (0.45-1.06) | 0.88  (0.61-1.26) | 0.89  (0.63-1.27) | 0.82  (0.53-1.27) | 0.73  (0.51-1.05) | 0.99  (0.68-1.45) | 1.00  (0.67-1.50) | 0.78  (0.53-1.14) | 0.86  (0.59-1.25) | 0.82  (0.57-1.19) | 1.12  (0.79-1.59) | 0.95  (0.64-1.39) | 0.80  (0.49-1.30) |  |
| School building 1800 MHz | 0.214 | **0.041** | **0.029** | 0.465 | 0.154 | 0.214 | 0.236 | 0.129 | 0.253 | 0.322 | 0.130 | 0.264 | 0.408 | 0.984 | 0.543 | 0.784 | 0.863 |  |
| 1^st^ quartile (≤0.0426 V/m) | 1 | 1 | 1 | 1 | 1 | 1 | 1 | 1 | 1 | 1 | 1 | 1 | 1 | 1 | 1 | 1 | 1 |  |
| 2^nd^ quartile (0.0427-0.0602 V/m) | 0.98  (0.66-1.45) | **0.68**  **(0.48-0.97)** | 0.81  (0.56-1.17) | 1.03  (0.72-1.49) | 1.57  (1.00-2.46) | 0.90  (0.63-1.29) | 0.77  (0.54-1.10) | 1.38  (0.91-2.07) | 0.88  (0.62-1.26) | 1.04  (0.71-1.52) | 0.88  (0.60-1.31) | 1.12  (0.77-1.63) | 0.93  (0.64-1.34) | 1.15  (0.80-1.65) | 0.98  (0.69-1.39) | 1.19  (0.81-1.75) | 1.04  (0.64-1.68) |  |
| 3^rd^ quartile (0.0603-0.190 V/m) | 1.10  (0.76-1.59) | 0.95  (0.67-1.33) | 0.94  (0.66-1.33) | 1.18  (0.83-1.68) | 1.04  (0.71-1.53) | 1.20  (0.84-1.70) | 0.84  (0.59-1.19) | 1.25  (0.83-1.86) | 1.16  (0.82-1.64) | 1.06  (0.74-1.51) | 0.83  (0.55-1.24) | 0.97  (0.67-1.41) | 0.79  (0.54-1.14) | 1.19  (0.83-1.68) | 1.12  (0.80-1.59) | 1.15  (0.79-1.67) | 0.96  (0.59-1.54) |  |
| 4^th^ quartile (≥0.191 V/m) | 0.87  (0.64-1.19) | 0.79  (0.58-1.06) | 0.88  (0.65-1.19) | 1.09  (0.81-1.47) | 0.94  (0.68-1.32) | 0.86  (0.64-1.16) | 0.88  (0.65-1.19) | **1.46**  **(1.03-2.07)** | 0.97  (0.72-1.31) | 1.12  (0.82-1.52) | 1.12  (0.80-1.56) | 0.94  (0.68-1.30) | 1.00  (0.73-1.37) | 1.19  (0.88-1.62) | 1.28  (0.95-1.72) | 1.12  (0.80-1.55) | 1.24  (0.81-1.89) |  |
| School garden RF | 0.905 | 0.787 | 0.880 | 0.635 | 0.802 | 0.101 | 0.623 | 0.841 | 0.148 | 0.116 | 0.268 | 0.522 | 0.659 | 0.449 | 0.420 | 0.410 | 0.180 |  |
| 1st quartile (≤0.426 V/m) | 1 | 1 | 1 | 1 | 1 | 1 | 1 | 1 | 1 | 1 | 1 | 1 | 1 | 1 | 1 | 1 | 1 |  |
| 2nd quartile (0.427-0.602 V/m) | 0.91  (0.56-1.45) | 1.08  (0.72-1.62) | 0.94  (0.61-1.47) | 0.66  (0.43-1) | 0.98  (0.57-1.67) | 0.96  (0.64-1.44) | 0.97  (0.65-1.46) | 0.93  (0.55-1.58) | 1.05  (0.70-1.58) | 1.10  (0.71-1.71) | 1.03  (0.65-1.63) | 0.71  (0.45-1.11) | 0.87  (0.56-1.36) | 0.66  (0.44-1.01) | 0.61  (0.41-0.92) | 0.68  (0.43-1.08) | 0.53  (0.27-1.06) |  |
| 3rd quartile (0.603-0.954 V/m) | 0.76  (0.55-1.06) | 1.14  (0.85-1.52) | 0.94  (0.69-1.27) | 1.03  (0.76-1.39) | 0.91  (0.64-1.31) | 0.83  (0.62-1.12) | 1.00  (0.75-1.34) | 1.13  (0.80-1.59) | 0.76  (0.56-1.01) | 0.87  (0.63-1.18) | 1.33  (0.96-1.84) | 0.90  (0.66-1.22) | 0.96  (0.71-1.30) | 0.89  (0.66-1.20) | 0.90  (0.68-1.21) | 1.10  (0.80-1.51) | 0.81  (0.54-1.21) |  |
| 4th quartile (≥0.955 V/m) | 1.08  (0.79-1.47) | 0.99  (0.75-1.31) | 0.92  (0.69-1.23) | 0.83  (0.62-1.11) | 0.95  (0.68-1.32) | 0.86  (0.65-1.14) | 0.87  (0.66-1.16) | 0.92  (0.66-1.30) | 0.85  (0.64-1.13) | 0.84  (0.63-1.13) | 1.11  (0.80-1.53) | 0.99  (0.73-1.34) | 1.14  (0.85-1.54) | 1.01  (0.76-1.34) | 0.94  (0.71-1.25) | 1.08  (0.80-1.47) | 0.90  (0.61-1.34) |  |
| School garden 3G | 0.861 | 0.549 | 0.969 | 0.353 | 0.144 | **0.011** | 0.907 | 0.532 | 0.128 | 0.164 | 0.098 | 0.660 | 0.895 | 0.488 | 0.566 | 0.456 | **0.014** |  |
| 1st quartile (≤0.00954 V/m) | 1 | 1 | 1 | 1 | 1 | 1 | 1 | 1 | 1 | 1 | 1 | 1 | 1 | 1 | 1 | 1 | 1 |  |
| 2nd quartile (0.00955-0.0151 V/m) | 0.82  (0.56-1.19) | 0.86  (0.62-1.19) | 0.89  (0.63-1.26) | 0.87  (0.62-1.23) | 0.84  (0.56-1.27) | 0.96  (0.69-1.34) | 0.93  (0.67-1.30) | 1.24  (0.84-1.82) | 0.96  (0.69-1.34) | 0.90  (0.63-1.29) | 0.98  (0.68-1.41) | 0.94  (0.66-1.32) | 0.97  (0.69-1.36) | 0.87  (0.62-1.21) | 0.82  (0.60-1.13) | 1.02  (0.71-1.47) | 0.66  (0.42-1.03) |  |
| 3rd quartile (0.0152-0.213 V/m) | 0.92  (0.62-1.37) | 1.03  (0.73-1.47) | 0.73  (0.51-1.06) | 0.91  (0.63-1.32) | 0.97  (0.63-1.51) | 0.94  (0.66-1.36) | 1.09  (0.77-1.56) | 0.86  (0.56-1.31) | 0.89  (0.62-1.27) | 0.93  (0.63-1.36) | 1.19  (0.81-1.76) | 0.88  (0.60-1.28) | 0.89  (0.61-1.29) | 1.02  (0.71-1.46) | 1.15  (0.81-1.63) | 1.30  (0.89-1.90) | 0.84  (0.53-1.32) |  |
| 4th quartile (≥0.214 V/m) | 1.03  (0.68-1.57) | 1.02  (0.71-1.47) | 1.03  (0.70-1.52) | 0.93  (0.63-1.35) | 0.73  (0.46-1.14) | 0.78  (0.54-1.12) | 1.07  (0.74-1.54) | 1.07  (0.69-1.66) | 0.85  (0.59-1.23) | 0.84  (0.57-1.24) | 1.15  (0.76-1.72) | 1.02  (0.69-1.51) | 1.07  (0.73-1.57) | 0.94  (0.65-1.36) | 0.80  (0.55-1.15) | 1.20  (0.80-1.79) | 0.66  (0.39-1.12) |  |
| School garden 900 MHz | 0.332 | 0.170 | 0.214 | 0.999 | 0.950 | 0.504 | 0.123 | 0.915 | 0.201 | 0.154 | 0.745 | 0.263 | 0.971 | 0.580 | 0.904 | 0.844 | 0.558 |  |
| 1st quartile (≤0.024 V/m) | 1 | 1 | 1 | 1 | 1 | 1 | 1 | 1 | 1 | 1 | 1 | 1 | 1 | 1 | 1 | 1 | 1 |  |
| 2nd quartile (0.025-0.170 V/m) | **0.69**  **(0.51-0.94)** | 0.93  (0.70-1.23) | 0.81  (0.61-1.09) | 0.98  (0.74-1.31) | 0.91  (0.65-1.26) | 0.95  (0.72-1.26) | 0.78  (0.59-1.03) | 1.34  (0.95-1.89) | 1.04  (0.79-1.39) | 0.99  (0.74-1.33) | 0.91  (0.66-1.27) | 0.81  (0.60-1.10) | 0.84  (0.62-1.13) | 0.97  (0.72-1.29) | 0.87  (0.66-1.16) | 0.85  (0.62-1.16) | 0.80  (0.52-1.24) |  |
| 3rd quartile (0.171-0.302 V/m) | **0.64**  **(0.46-0.89)** | 0.99  (0.74-1.32) | 0.82  (0.61-1.11) | 1.12  (0.83-1.51) | 0.93  (0.65-1.33) | 0.82  (0.61-1.10) | 0.83  (0.62-1.10) | 1.40  (0.99-1.99) | 0.77  (0.57-1.02) | 0.86  (0.63-1.17) | 1.24  (0.90-1.71) | 0.86  (0.64-1.17) | 0.95  (0.70-1.28) | 1.06  (0.79-1.41) | 1.15  (0.87-1.53) | 1.12  (0.82-1.54) | 1.07  (0.71-1.6) |  |
| 4th quartile (≥0.303 V/m) | 0.82  (0.55-1.22) | 0.73  (0.51-1.04) | 0.82  (0.56-1.18) | 0.79  (0.55-1.14) | 0.82  (0.54-1.25) | 0.88  (0.62-1.26) | 0.70  (0.49-1.00) | 1.08  (0.70-1.67) | 0.89  (0.62-1.27) | 0.77  (0.53-1.12) | 1.18  (0.80-1.74) | 0.87  (0.59-1.27) | 1.10  (0.76-1.59) | 1.02  (0.71-1.46) | 1.06  (0.75-1.51) | 0.97  (0.66-1.43) | 0.77  (0.46-1.29) |  |
| School garden 1800 MHz | 0.132 | 0.280 | 0.933 | 0.455 | 0.067 | 0.065 | 0.880 | 0.694 | 0.598 | 0.403 | **0.038** | 0.485 | 0.990 | 0.568 | 0.431 | 0.899 | 0.129 |  |
| 1st quartile (≤0.0426 V/m) | 1 | 1 | 1 | 1 | 1 | 1 | 1 | 1 | 1 | 1 | 1 | 1 | 1 | 1 | 1 | 1 | 1 |  |
| 2nd quartile (0.0427-0.0535 V/m) | 0.81  (0.54-1.23) | 0.93  (0.64-1.35) | 1.17  (0.79-1.71) | 1.45  (0.99-2.12) | 1.15  (0.73-1.80) | 0.92  (0.63-1.33) | 0.99  (0.68-1.43) | 1.38  (0.90-2.13) | 1.21  (0.84-1.76) | 1.11  (0.75-1.64) | 0.81  (0.53-1.23) | 1.15  (0.78-1.69) | 0.94  (0.64-1.39) | 1.09  (0.75-1.59) | 1.02  (0.71-1.47) | 1.05  (0.70-1.57) | 0.97  (0.58-1.62) |  |
| 3rd quartile (0.0536-0.170 V/m) | 0.88  (0.63-1.22) | 1.07  (0.80-1.44) | 0.97  (0.72-1.32) | 1.06  (0.78-1.42) | 0.96  (0.67-1.37) | 0.92  (0.69-1.24) | 1.08  (0.81-1.45) | 1.34  (0.94-1.92) | 1.16  (0.87-1.56) | 1.23  (0.90-1.68) | 1.11  (0.80-1.55) | 0.89  (0.65-1.22) | 1.02  (0.75-1.38) | 1.05  (0.78-1.42) | 1.01  (0.75-1.34) | 1.09  (0.79-1.51) | 1.01  (0.66-1.56) |  |
| 4th quartile (≥0.171 V/m) | 0.88  (0.63-1.24) | 1.24  (0.92-1.67) | 1.01  (0.74-1.38) | 1.08  (0.80-1.47) | 0.88  (0.62-1.26) | 0.85  (0.63-1.14) | 1.16  (0.86-1.56) | 0.94  (0.64-1.36) | 1.04  (0.77-1.41) | 0.98  (0.72-1.33) | 1.27  (0.91-1.77) | 0.98  (0.71-1.35) | 1.07  (0.79-1.47) | 1.03  (0.76-1.40) | 0.93  (0.70-1.26) | 1.08  (0.78-1.51) | 0.83  (0.53-1.30) |  |

**Bold, black ORs:** Significant ORs

**Bold, red p:** Significant p trends

*The category “does not know” (n=1171) excluded from the analysis

**Pearson Chi-square (not p for trend due to number of categories to compare)
